# Correction: Huang et al. PAICS/DYRK3 Multienzyme Interactions as Coregulators of Purinosome Formation and Metabolism on Radioresistance in Oral Squamous Cell Carcinoma. *Int. J. Mol. Sci.* 2023, *24*, 17346

**DOI:** 10.3390/ijms27094007

**Published:** 2026-04-30

**Authors:** Chin-Sheng Huang, Ming-Shou Hsieh, Vijesh Kumar Yadav, Yang-Che Wu, Shao-Cheng Liu, Chi-Tai Yeh, Mao-Suan Huang

**Affiliations:** 1 Department of Dentistry, Taipei Medical University-Shuang Ho Hospital, New Taipei City 235, Taiwan; 09520@s.tmu.edu.tw (C.-S.H.); dr7d9eddd@gmail.com (M.-S.H.); vijeshp2@gmail.com (V.K.Y.); yangchewu@tmu.edu.tw (Y.-C.W.); 2 School of Dentistry, College of Oral Medicine, Taipei Medical University, Taipei City 110, Taiwan; 3 Department of Dentistry and Oral Health, Taipei Medical University-Shuang Ho Hospital, New Taipei City 235, Taiwan; 4 Department of Otolaryngology-Head and Neck Surgery, Tri-Service General Hospital, National Defense Medical Center, Taipei City 114, Taiwan; m871435@mail.ndmctsgh.edu.tw; 5 Department of Medical Research & Education, Taipei Medical University-Shuang Ho Hospital, New Taipei City 235, Taiwan; 6 Continuing Education Program of Food Biotechnology Applications, College of Science and Engineering, National Taitung University, Taitung 950, Taiwan

In the original publication [1], there was a mistake in the Institutional Review Board Statement and Informed Consent Statement of this article. The Institutional Review Board Number Statement in Section 4, Materials and Methods, also needs to be corrected. The corrected version appears below. The authors state that the scientific conclusions are unaffected. This correction was approved by the Academic Editor. The original publication has also been updated.

**4. Materials and Methods**

*4.1. Collection of Clinical Specimens and Creation of In Vitro Cell Model of Patient-Derived Primary Cells*

Obtain tissue samples from 50 individuals with radiation therapy oral squamous cell carcinoma (OSCC) at Tri-Service General Hospital (TSGH). The research, which focuses on studying the expression of DYRK3/PAICS upstream and downstream targets, commences after receiving approval from the institutional review board (IRB No. 2-108-05-124) of Tri-Service General Hospital and adhering to the ethical guidelines outlined in the Declaration of Helsinki for biomedical research. To assess the expression of DYRK3 in recurrent OSCC, we construct a tissue array comprising samples from a total of 50 subjects. This array will consist of 20 non-recurrence tissues and 30 radio-resistance tissues obtained from patients with radioresistant and metastatic OSCC. Immunohistochemical (IHC) staining will be conducted on the tissue array using an antibody specifically targeting DYRK3 (at a concentration of 1:400, ab96617, Abcam, Greater Boston, MA, USA). The staining process will follow the standard IHC protocol, including the use of mouse immunoglobulin G (IgG) as a negative control at a comparable dilution. To establish primary cells derived from the patients’ tumors, small pieces of tumor tissue (measuring 2 mm^3^) will be surgically obtained during the procedure. These tissue fragments, known as explants, will be placed in a 24-well plate coated with fetal bovine serum. Essential nutrients will be supplemented to facilitate optimal growth. The medium volume will be adjusted to ensure that the explants adhere to the bottom of the plate and do not float. Regular monitoring of the explants will take place, and any explants exhibiting fibroblast growth will be discarded, retaining only those with a single layer of epithelium. Once the epithelial layer forms a distinct halo, the explants will be transferred to a new Petri dish for further passages. This continuous process of passage will be repeated to acquire primary tumor cell lines.

**Institutional Review Board Statement**: All animal studies were performed in accordance with protocols approved by the Institutional Animal Care and Use Committee (IACUC) at Taipei Medical University, Taipei, Taiwan (approval number: LAC-2022-0467). All experiments complied with the guidelines provided in the Guide for the Care and Use of Laboratory Animals published by the National Academies of Science, Engineering, and Medicine. This study was conducted in full accordance with the ethical principles outlined in the Declaration of Helsinki for biomedical research involving human subjects. Ethical approval was obtained from the Institutional Review Board (IRB) of Tri-Service General Hospital (IRB No. 2-108-05-124).

**Informed Consent Statement**: Informed consent was obtained from all subjects involved in the study. 
