# Supplementary material for: PAICS/DYRK3 Multienzyme Interactions as Coregulators of Purinosome Formation and Metabolism on Radioresistance in Oral Squamous Cell Carcinoma
Source: Int J Mol Sci. 2023 Dec 11;24(24):17346. doi: 10.3390/ijms242417346 (PMC10744311; doi:10.3390/ijms242417346)
Supplement: Supplementary file 1 [file ijms-24-17346-s001.zip › ijms-2702323-supplementary.pdf]

SUPPLEMENTARY INFORMATION

PAICS/DYRK3 Multienzyme Interactions as Coregulators of Purinosome Formation and Metabolism on Radioresistance in Oral Squamous Cell Carcinoma

Chin-Sheng Huang 1,2,†, Ming-Shou Hsieh 1,2,†, Vijesh Kumar Yadav 1,2, Yang-Che Wu 1,2,3, Shao-Cheng Liu 4, Chi-Tai Yeh 5,6,* and Mao-Suan Huang 1,2,*

1 Department of Dentistry, Taipei Medical University-Shuang Ho Hospital, New Taipei City 235, Taiwan

2 School of Dentistry, College of Oral Medicine, Taipei Medical University, Taipei City 110, Taiwan

3 Department of Dentistry and Oral Health, Taipei Medical University-Shuang Ho Hospital, New Taipei City 235, Taiwan

4 Department of Otolaryngology-Head and Neck Surgery, Tri-Service General Hospital, National Defense Medical Center, Taipei City 114, Taiwan

5 Department of Medical Research & Education, Taipei Medical University-Shuang Ho Hospital, New Taipei City 235, Taiwan

6 Continuing Education Program of Food Biotechnology Applications, College of Science and Engineering, National Taitung University, Taitung 950, Taiwan

* Correspondence: ctyeh@s.tmu.edu.tw (C.-T.Y.); hms4837@tmu.edu.tw (M.-S.H.); Tel.: +886-2-2490088 (ext. 8881) (C.-T.Y. & M.-S.H.); Fax: +886-2-2248-0900 (C.-T.Y. & M.-S.H.)

† These authors contributed equally to this work

Supplementary Table S1. Q-PCR primer list.

Primer	forward	reverse
DYRK3	CTGCAATCCTTCTGAACCACCTC	CTACCTTCATCTCACCTCCATCC
PAICS	ACCACCTGGAAGGAAAAGCTGC	CGGTGCAATGAAAGCTGTCTCC
PPAT	GCGATTGAAGCACCTGTGGATG	CGGTTTTTACACAGCACCTCCAC
GIT2	GAGTTTGCCACGCTGGTCATTG	GACTCTCAACGCTGTGCTGGTT
ATM	TGTTCCAGGACACGAAGGGAGA	CAGGGTTCTCAGCACTATGGGA
β-Actin	CACCATTGGCAATGAGCGGTTC	AGGTCTTTGCGGATGTCCACGT

Supplementary Table S2. Antibody list.

Antibody	catalog number	source
DYRK3 mAb	ab96617	Abcam
PAICS mAb	ab151472	Abcam
PPAT mAb	ab248259	Abcam
Glut-1 mAb	ab115730	Abcam
β-Actin mAb	#4967	Cellsignaling

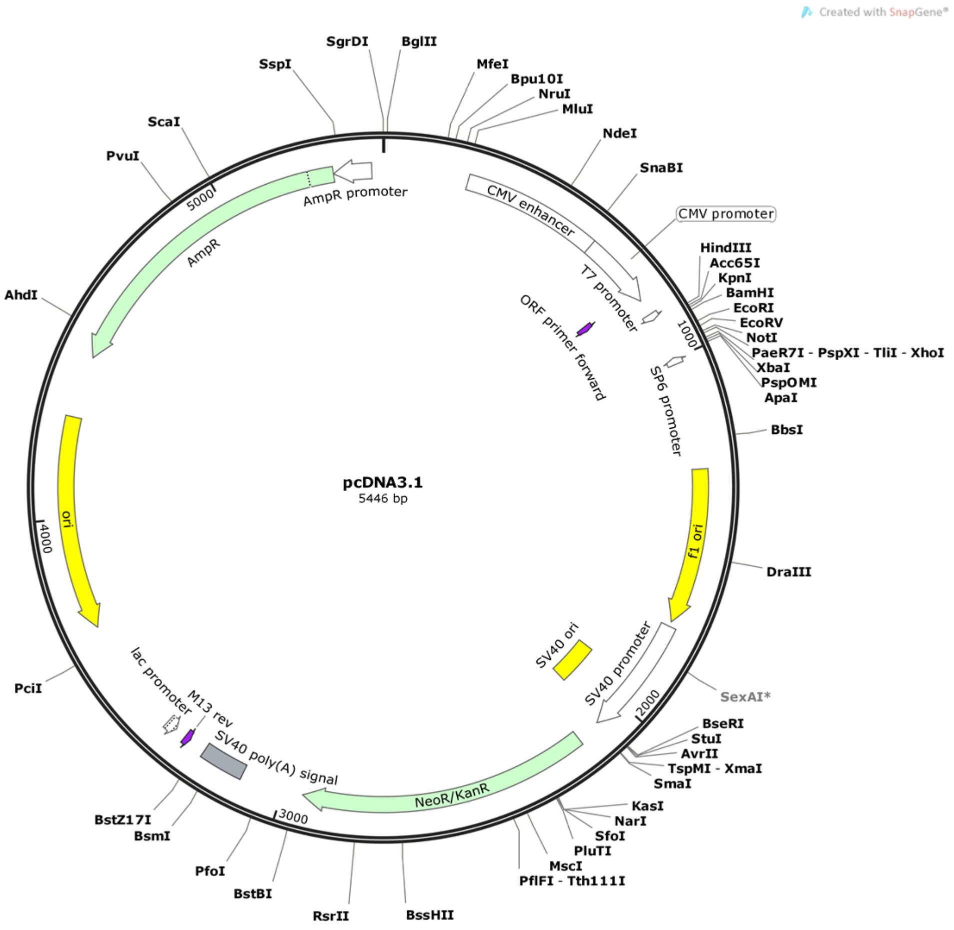

Primer	Sequence
DYRK3-ECOR1-forward	AATTGAATTCCTGCAATCCTTCTGAACCACCTC
DYRK3-Xba1-reversed	AATTTCTAGACTACCTTCATCTCACCTCCATCC

Supplementary Figure S1: Overexpression of DYRK3 plasmid backbone and primer design.

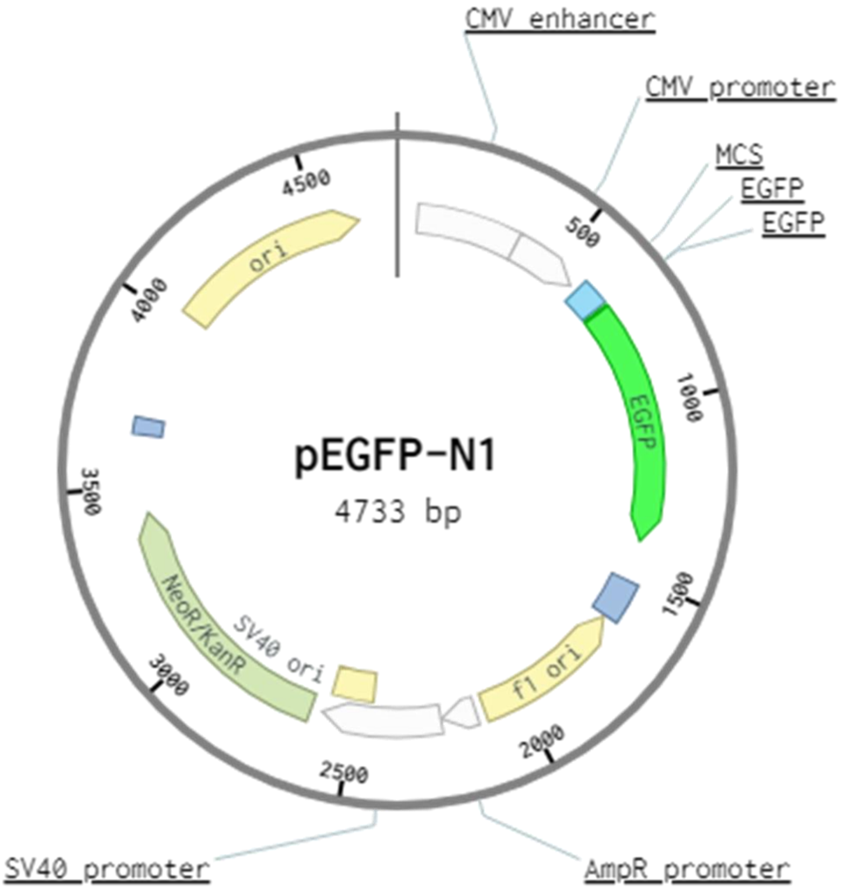

Primer	Sequence
PAICS-ECOR1-forward	AATTGAATTCACCACCTGGAAGGAAAAGCTGC
PAICS-BamH1-reversed	AATTGGATCCCGGTGCAATGAAAGCTGTCTCC
PPAT-ECOR1-forward	AATT GAATTCGCGATTGAAGCACCTGTGGATG
PPAT-BamH1-reversed	AATT GGATCCCGGTTTTTACACAGCACCTCCAC

Supplementary Figure S2: The fluorescent protein fusion vector PAICS-EGFP-N1 and PPAT-EGFP-N1.
